# PEGylated versus non-PEGylated magnetic nanoparticles as camptothecin delivery system

**DOI:** 10.3762/bjnano.5.144

**Published:** 2014-08-19

**Authors:** Paula M Castillo, Mario de la Mata, Maria F Casula, José A Sánchez-Alcázar, Ana P Zaderenko

**Affiliations:** 1 INSTM and Dipartimento di Scienze Chimiche e Geologiche. Università di Cagliari, Italy; 2 Departamento de Sistemas Físicos, Químicos y Naturales. Universidad Pablo de Olavide, Sevilla, Spain; 3 Centro Andaluz de Biología del Desarrollo (CABD-CSIC-Universidad Pablo de Olavide), Sevilla, Spain

**Keywords:** camptothecin, cancer therapy, iron oxide superparamagnetic nanoparticles, polyethylene glycol

## Abstract

Camptothecin (CPT; (*S*)-(+)-4-ethyl-4-hydroxy-1*H*-pyrano[3',4':6,7]indolizino[1,2-*b*]quinoline-3,14-(4*H*,12*H*)-dione) is a highly cytotoxic natural alkaloid that has not yet found use as chemotherapeutic agent due to its poor water-solubility and chemical instability and, as a consequence, no effective administration means have been designed. In this work, camptothecin has been successfully loaded into iron oxide superparamagnetic nanoparticles with an average size of 14 nm. It was found that surface modification of the nanoparticles by polyethylene glycol enables loading a large amount of camptothecin. While the unloaded nanoparticles do not induce apoptosis in the H460 lung cancer cell line, the camptothecin-loaded nanoparticle formulations exhibit remarkable pro-apoptotic activity. These results indicate that camptothecin retains its biological activity after loading onto the magnetic nanoparticles. The proposed materials represent novel materials based on naturally occurring bioactive molecules loaded onto nanoparticles to be used as chemotherapeutic formulations. The procedure seems apt to be extended to other active molecules extracted from natural products. In addition, these materials offer the potential of being further implemented for combined imaging and therapeutics, as magnetic nanoparticles are known to be multifunctional tools for biomedicine.

## Introduction

**Figure 1:**
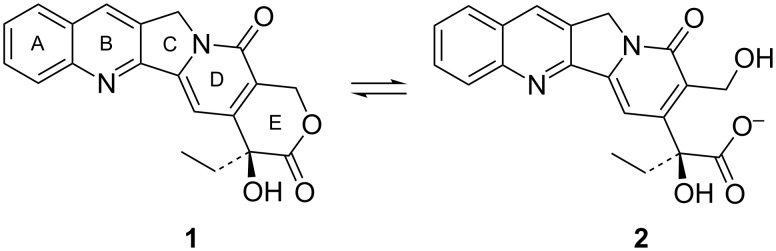
Molecular structure of (S)-(+)-camptothecin (**1**) and its inactive form (**2**) through lactone ring hydrolysis at physiological pH.

Camptothecin (CPT) is a quinoline based alkaloid, which exhibits a potent cytotoxic activity against a broad spectrum of tumours [1–3]. While most antineoplastic agents inhibit cancer cell proliferation by binding to DNA, CPT antitumor activity is due to inhibition of the nuclear enzyme topoisomerase I [4–5]. In spite of its potential as chemotherapeutic agent, CPT suffers from a reduced in vivo antitumor efficacy owing to its poor water-solubility and chemical instability (Figure 1). CPT-derivatives with improved solubility and stability have been developed; nevertheless their overall therapeutic impact is modest due to their lower activity when compared to CPT [6–7].

An additional drawback related to the use of chemotherapeutic drugs refers to their lack of selectivity and, consequently, to their undesirable side effects. Due to the combined impact of cancer together with adverse side effects of many conventional chemotherapeutic agents, a significant effort is devoted to the design of nanoparticle vectors for cancer therapy [8–10]. Concerning CPT, attempts to improve its solubility and stability by means of nano-formulations cover a wide range of organic nanomaterials [11–19]. Noticeably, a cyclodextrin-containing polymer–CPT nano-formulation is currently undergoing phase II clinical trials [20].

Superparamagnetic iron oxide nanoparticles (SPION) are particularly promising as delivery systems due to their low toxicity and their ability to be used both in cancer diagnosis and therapy [21–23]. SPION can be effectively used as contrast agents for magnetic resonance imaging [24–25], as carriers for chemotherapeutic drugs [26–28] and to destroy cancer cells by acting as heat mediators in hyperthermia treatments [29–31]. Despite their interest, few attempts to develop CPT-delivery systems based on SPION have been reported [32–34], and none of them examine their ability to adsorb CPT, nor takes advantage of the desirable properties offered by polyethylene glycol (PEG) as coating polymer in nano-formulations. PEG has been widely used in the formulation of nanoparticles for biomedical applications, both because of its biocompatibility and its effectiveness in *camouflaging* nanoparticles from opsonins [35]. Recently, it has been described that PEG coating further reduces SPION cytotoxicity [36]. Moreover, PEGylated CPT has demonstrated its capability to lock the CPT E ring in its desired active lactone configuration [37].

Herein, we report a simple method to synthesise PEG-coated ultrasmall magnetite (USM) nanoparticles, and we examine the ability of both, bare and PEGylated USM nanoparticles, to conjugate CPT.

## Findings

**Figure 2:**
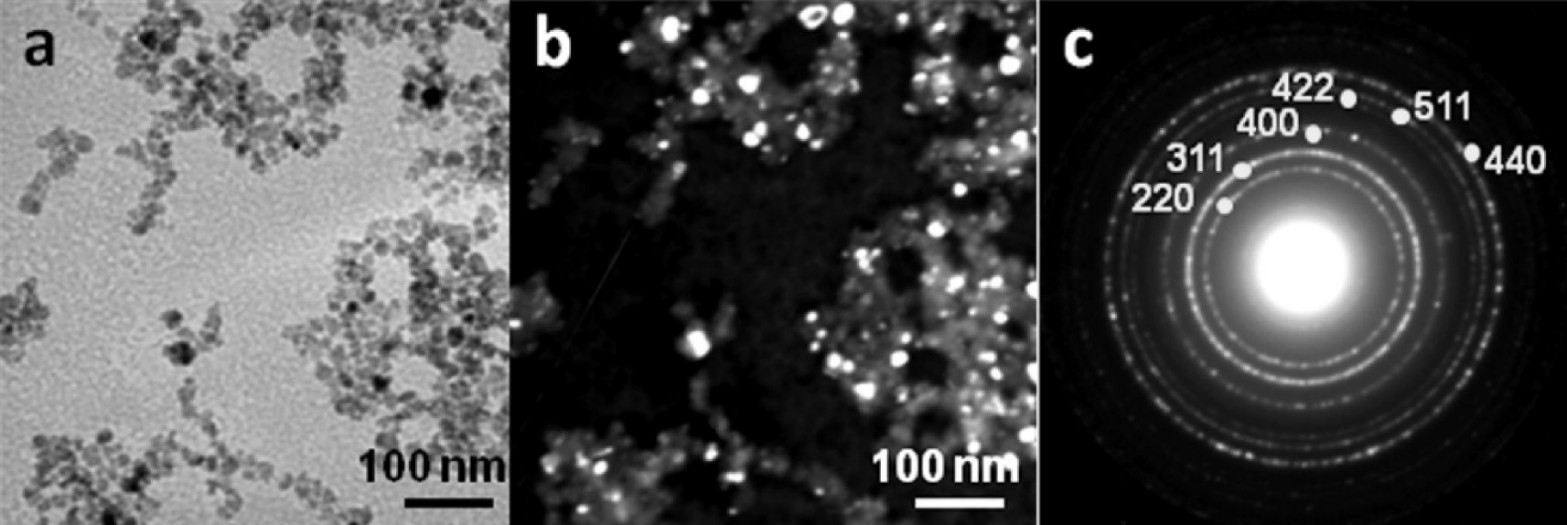
Bright field (a) and dark field (b) transmission electron microscopy (TEM) images and diffraction pattern (c) of USM nanoparticles.

USM nanoparticles were synthesised through an iron co-precipitation method under alkaline conditions as described by Bee et al. [38] with slight modifications [39] (for detailed descriptions of the experimental procedures see Supporting Information File 1). As shown in Figure 2a, the USM nanoparticles are nearly spherical, with an average particle diameter of 14.0 nm and a standard deviation of 2.0 nm, and monocrystalline (Figure 2b). The electron diffraction pattern corresponds to the spinel iron oxide nanocrystalline phase (Figure 2c).

**Figure 3:**
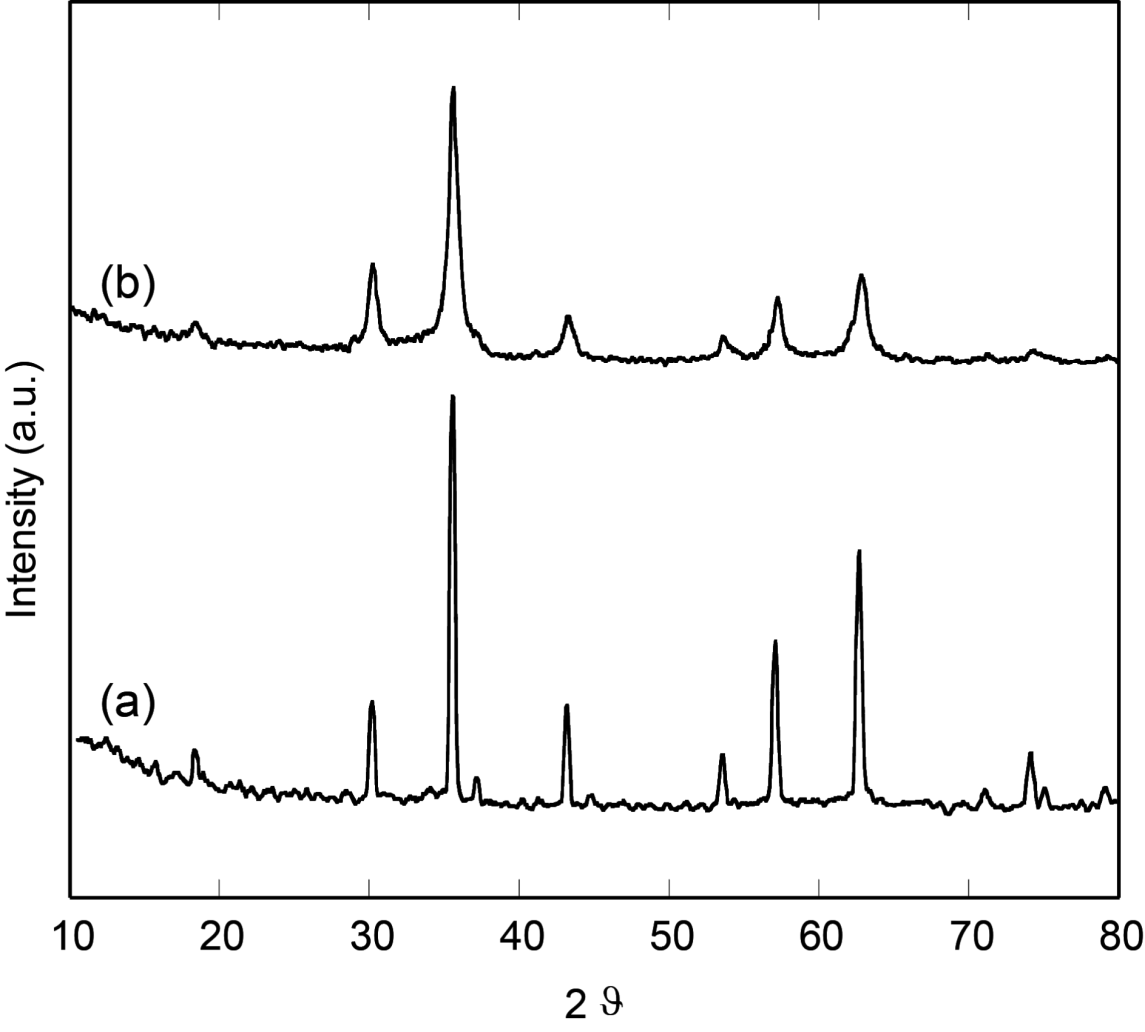
XRD spectrum of standard magnetite (a) and USM nanoparticles (b).

Further insights into the crystalline structure were obtained from X-ray diffraction (XRD) patterns. Figure 3 reports the XRD pattern of our USM nanoparticles compared to a reference of a commercial magnetite standard. Although it is not possible to unambiguously ascribe the obtained pattern for USM nanoparticles to magnetite rather than to the isostructural spinel ferric iron oxide (maghemite), the XRD sample of the USM nanoparticles is consistent with the formation of magnetite. The peak broadening of the XRD pattern of the USM sample is in agreement with its nanocrystalline form. In particular, the average size determined by line profile analysis is 11.0 ± 1.0 nm, in good agreement with the TEM data.

The USM sample readily responds to an external magnet and the main magnetic parameters, as derived by SQUID magnetometry characterisation, are summarised in Table 1. ZFC-FC magnetisation curves indicate that the USM nanoparticles exhibit a superparamagnetic behaviour and that most of the particles are blocked at room temperature, the blocking and maximum temperatures being out of the investigated range. The saturation magnetisation (*M*_sat_) as obtained from the hysteresis curve collected at 5 K was measured to be around 72 emu·g^−1^ which is close to the value of bulk magnetite and maghemite (ca. 90 and 80 emu·g^−1^, respectively). A decrease in saturation magnetisation values is often observed in nanoparticles and ascribed both to the effect of surface atoms and to a reduced crystallinity compared to their bulk counterparts [40]. As shown by the very low residual magnetisation value as compared to the saturation magnetisation, the sample at 5 K is nearly saturated at high fields and is mainly in the blocked state.

**Table 1:** Main magnetic parameters as derived for the USM sample by SQUID magnetometry: Maximum and separation temperatures (*T*_max_ and *T*_sep_) as obtained by ZFC-FC magnetization curves; coercive field (*H*_c_), saturation magnetization (*M*_sat_); residual magnetization (*M*_r_) and residual versus saturation magnetization values (*M*_r_/*M*_sat_) as obtained by the hysteresis curve collected at 5 K.

*T*_max_ (K)	*T_sep_* (K)	*H*_c_ (Oe)	*M*_r_ (emu/g)	*M*_sat_ (emu/g)	*M*_r_/*M*_sat_

>325	>325	139.218	6.238	72.402	0.086

**Scheme 1:**
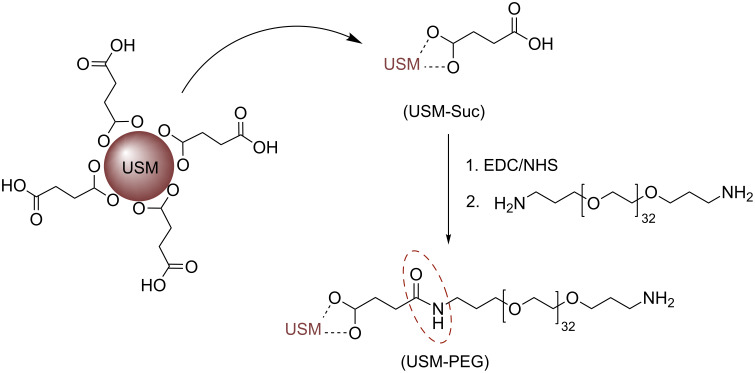
Carbodiimide-mediated covalent attachment of PEG to USM-Suc.

The obtained USM nanoparticles were PEGylated according to the strategy depicted in Scheme 1. In a typical PEGylation procedure the surface of USM nanoparticles was first coated with succinic acid, in order to allow for the subsequent covalent linkage of bis(3-aminopropyl)-terminated poly(ethylene glycol) through carbodiimide chemistry.

It is known that the use of succinic acid as stabilizing agent during the synthesis of magnetite nanoparticles decorates their surface with acid molecules. Nevertheless, the amount of succinic acid that has been attached to the surface is low, as can be judged from the IR data available [41]. Given that the amount of succinic linkers on the nanoparticle surface limits the uptake of PEG, it is crucial to ensure higher amounts of carboxylic groups on the nanoparticle surface. In order to increase the acid coating, we have investigated a different approach which relies on the direct incubation of previously synthesized magnetite nanoparticles with the acid in aqueous medium, instead of including it into the co-precipitation reaction medium. By this approach we were able to obtain USM-Suc nanoparticles whose surface is densely covered with carboxylic acid.

**Figure 4:**
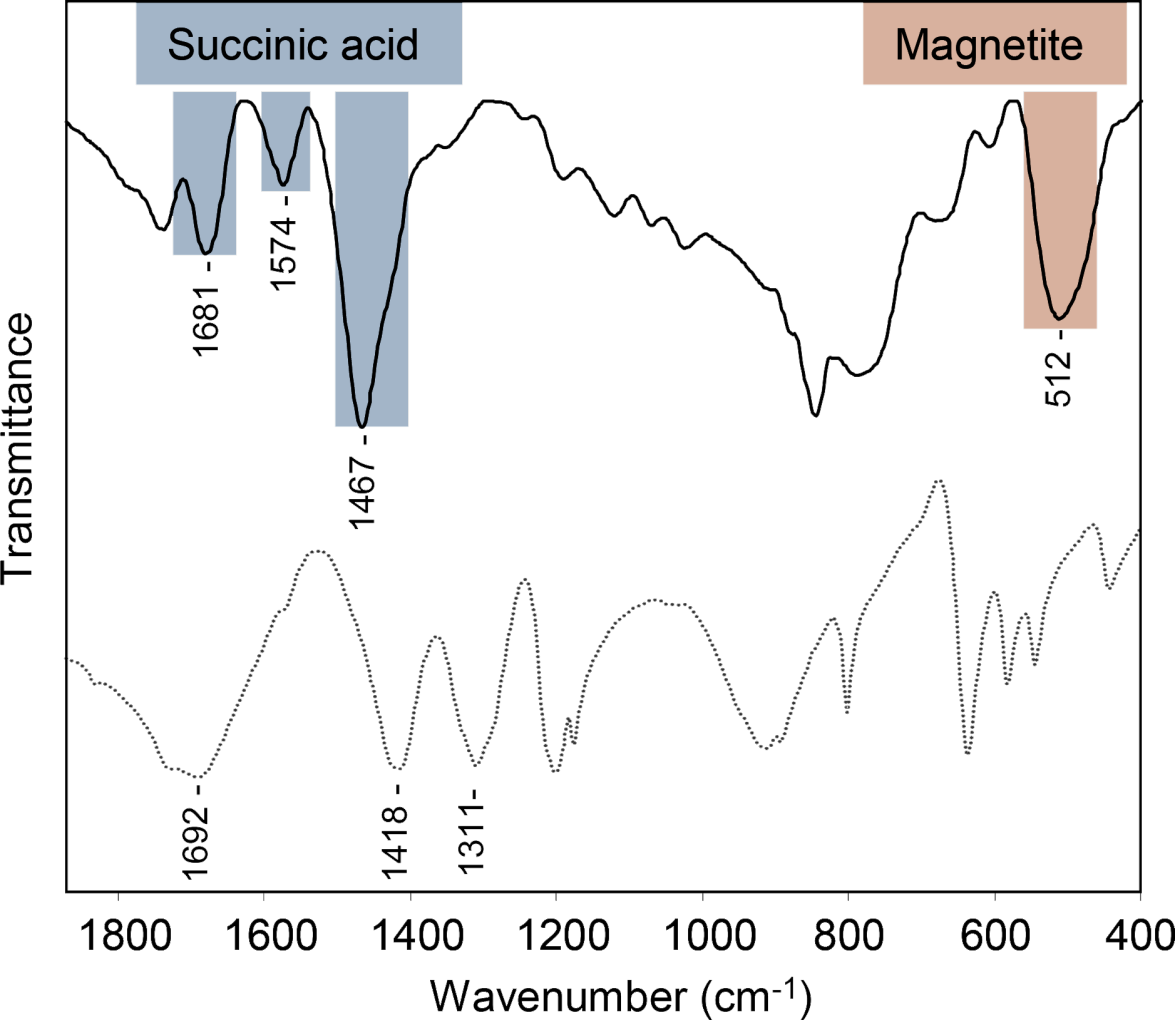
FTIR spectrum of USM-Suc nanoparticles (solid line) and succinic acid (dotted line) as a reference. Highlighted signals are discussed in the text.

Figure 4 shows the FTIR spectra of USM-Suc and succinic acid in the most commonly used mode: transmittance. However, in order to make a quantitative comparison between peak areas, the corresponding absorbance spectra were used. The comparison between the areas of two peaks, one of succinic acid at about 1467 cm^−1^, made up from a weaker contribution from skeletal carbon in succinic acid (CH_2_ scissoring, 1419 cm^−1^) and the symmetric stretching of the carboxylate group (around 1467 cm^−1^, total area = 11.2), and the other at 512 cm^−1^ that is dependent on the amount of USM nanoparticles (δFe-O, area = 6.6) yields an area ratio of 1.7. In comparison, the skeletal carbon signal in [41] is virtually absent from the spectrum. According to our FTIR data we may reasonably assume that the high succinic acid surface coating will translate into a high coating of PEG in the subsequent functionalization step. The FTIR spectrum, through the aforementioned symmetric stretching of the carboxylate group plus the asymmetric stretching at 1574 cm^−1^ supports that binding to the magnetic core occurs through carboxylate groups, whereas free acid groups are also detected in the spectrum (around 1700 cm^−1^) that must be located on the outside of the particles, as illustrated in Scheme 1.

In order to covalently attach the amino-terminated polymer PEG onto the nanoparticle surface, a water soluble carbodiimide (*N*-(3-dimethylaminopropyl)-*N*′-ethylcarbodiimide hydrochloride, EDC) was used that conjugates the primary amine group of PEG with the free carboxylic end group of the USM-Suc nanoparticles. EDC was used in tandem with *N*-hydroxysuccinimide (NHS) to improve the water stability of the carboxylic acid-activated intermediate, and excess PEG was used for maximum coating of nanoparticles [42].

**Figure 5:**
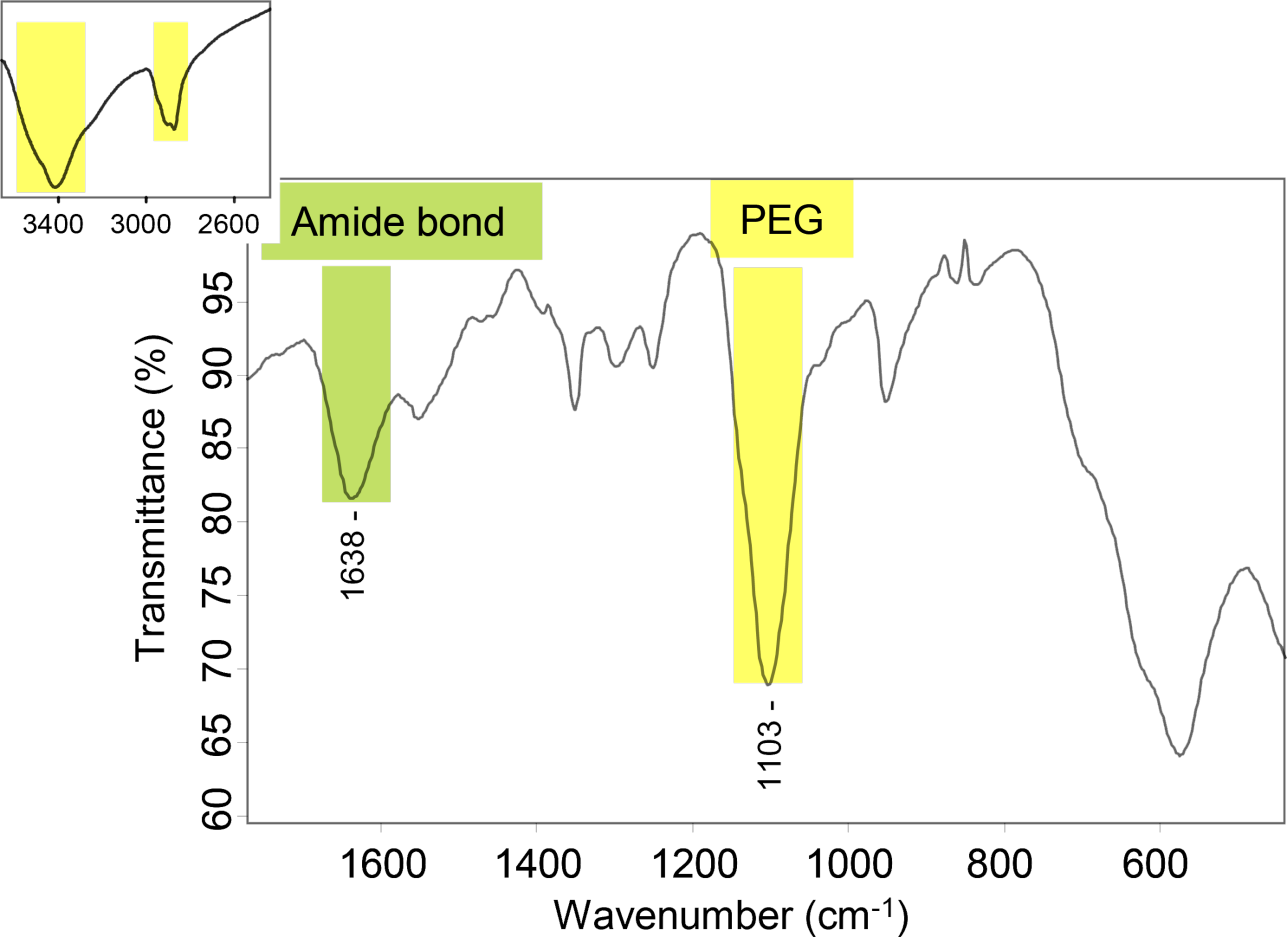
FTIR spectrum of USM-PEG nanoparticles. Highlighted signals are discussed in the text.

The comparison between the FTIR spectra of USM-Suc and USM-PEG, Figure 4 and Figure 5 respectively, demonstrates that the coupling reaction was successful. The characteristic vibration of the succinic carboxylic acid group in USM-Suc (C=O stretching mode at 1700 cm^−1^), disappears in USM-PEG owing to its replacement by an amide bond (1638 cm^−1^). An even clearer proof of the functionalization is the appearance of intense characteristic bands of PEG at 2891 and 1103 cm^−1^, corresponding to the ether C–H and C–O–C stretching modes, respectively, and the band due to the remaining free primary amine of PEG covalently bonded to the surface of USM (N–H stretching) at 3419 cm^−1^.

It is noteworthy that electrophoretic techniques have also been developed in order to quantitatively determine the number of PEG molecules in gold and quantum dot-PEG conjugates [43].

Loading of CPT on USM and USM-PEG nanoparticles was performed by direct incubation of the drug in an aqueous solution of the nanoparticles to obtain the formulations USM[CPT] and USM-PEG[CPT], respectively, and their maximum loading capacity was calculated by Equation 1:

[1]
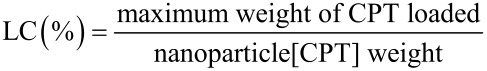


The loading capacity was obtained from UV assays according to the details given in Supporting Information File 1, and the results are summarized in Table 2. Both bare and PEGylated nanoparticles were able to take up a substantial amount of CPT. Although the loading capacity of USM bare nanoparticles was not as high as the PEGylated ones, it is still higher than that reported for other polymer-coated USM nanoparticles [33], exceeding even the loading capacity of the prodrug Prothecan (1.7% (w/w)) [44]. The extremely high loading capacity of USM-PEG can be attributed to the amphiphilic nature of PEG polymers. It is noteworthy that although PEG is commonly used in block copolymers as hydrophilic moiety, it is soluble in both water and organic solvents owing to the hydrophobic nature of the ethylene groups. Although few examples are known in the literature, PEG polymers have proven their ability to interact with hydrophobic drugs [45].

**Table 2:** Maximum loading capacities of USM[CPT] and USM-PEG[CPT] nanoparticles calculated according to Equation 1 and compared to [33].

this work	LC (%)	reference [33]	LC (%)

USM[CPT]	13.15 ± 0.05	CPT-loaded CS/Fe_3_O_4_	3.47 ± 0.09
USM-PEG[CPT]	26.11 ± 0.02	CPT-loaded OCMCS/Fe_3_O_4_	2.85 ± 0.05
		CPT-loaded NSOCMCS/Fe_3_O_4_	3.01 ± 0.06

**Figure 6:**
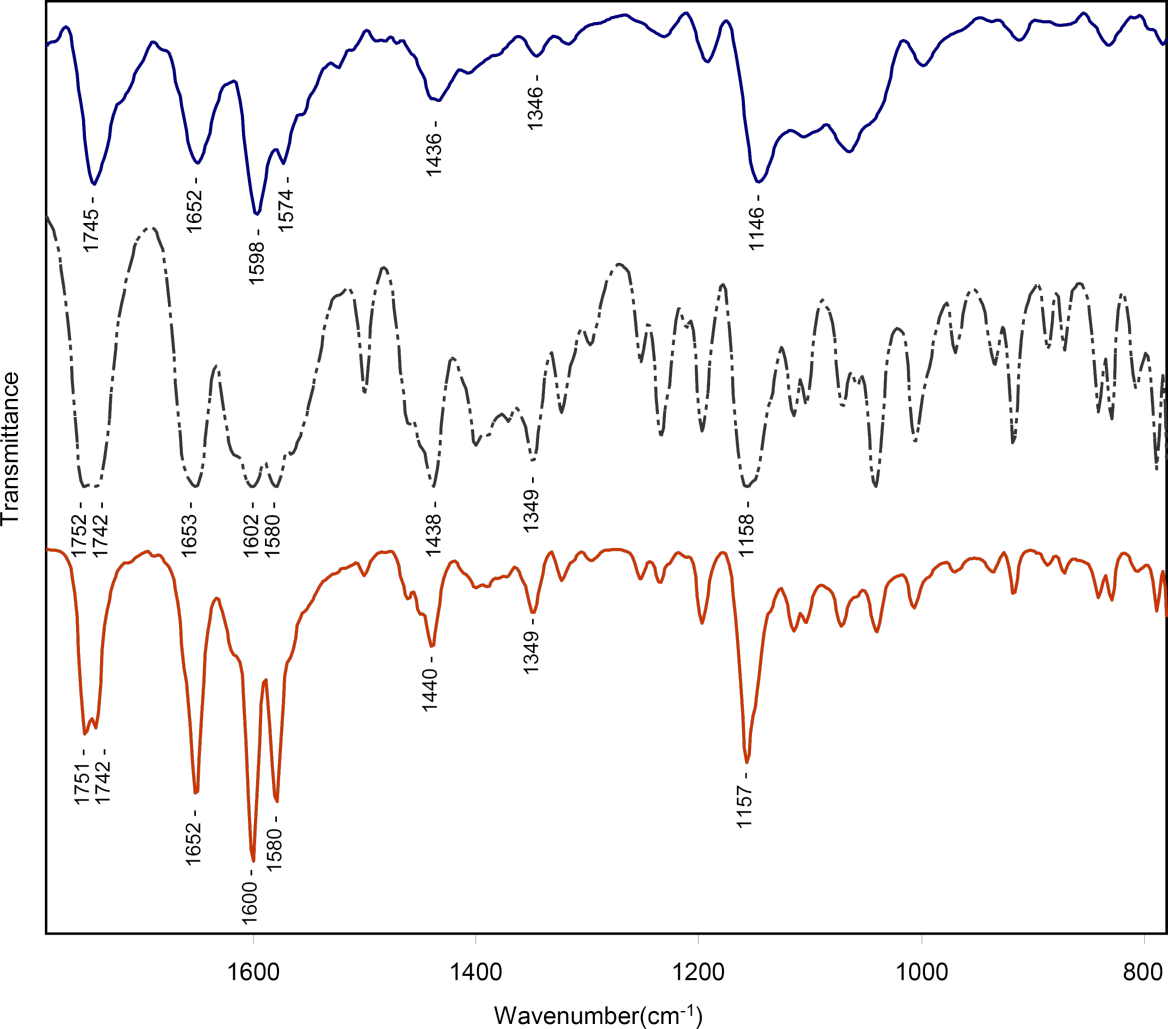
Infrared spectra of USM[CPT] (top, blue solid line), CPT (center, grey dotted line) and USM-PEG[CPT] (bottom, red solid line).

The chemical integrity of CPT in USM[CPT] and USM-PEG[CPT] was investigated by FTIR spectroscopy. As shown in Figure 6 the characteristic peaks of CPT are clearly present in both USM[CPT] and USM-PEG[CPT]. Although taken at a lower resolution, probably due to the lower concentration of CPT loaded in USM[CPT] when compared to USM-PEG[CPT], the USM[CPT] formulation possesses also the characteristic peaks of its CPT load. Differences in intensity and a shift are expected and obtained between the characteristic bands of CPT in USM[CPT] and USM-PEG[CPT], owing to the different nature of the nanoparticle surface, which results in different interactions with CPT. It is noteworthy that the characteristic lactone band at 1750 cm^−1^ (C=O stretching vibration of lactone) is retained in both USM[CPT] and USM-PEG[CPT] spectra, indicating that the chemical integrity of CPT in nanoconjugates is not affected during the formulation process.

**Figure 7:**
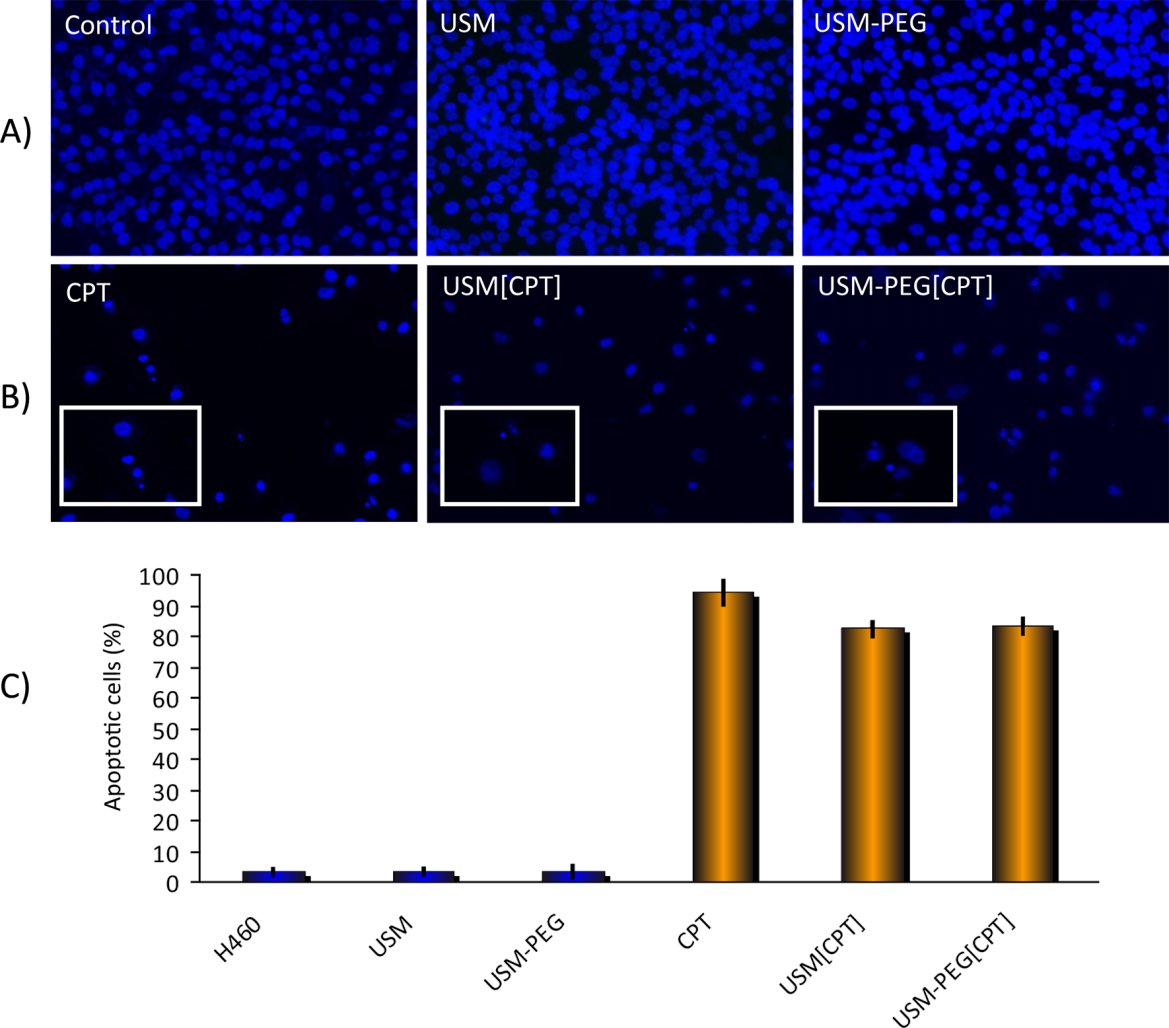
A: Fluorescence microscopy images of H460 cell cultures: control (left); with bare USM (middle); with PEGylated USM (right). B: Fluorescence microscopy images of the H460 cell culture loaded with: CPT (left); USM[CPT] formulation (middle); USM-PEG[CPT] formulation (right) and details of apoptotic nuclei (inserted). C: Percentage of apoptotic cell nuclei in H460 cell cultures: From left to right, control, with bare USM, with PEGylated USM, with CPT, with USM[CPT], USM-PEG[CPT]. Stock dispersions of USM[CPT] and USM-PEG[CPT] were prepared in PBS (pH 7.5) with a CPT concentration of 5 mM, and 1 µL of this dispersion was added to H460 cells previously cultured (confluent; 1mL final volume, 5 μM final CPT concentration). Cells with nanoparticles and references were incubated at 37 °C for 48 h. More than 100 cells were examined for each experimental condition. *P* < 0.05 significant differences with respect to control cells.

As chemical integrity of CPT is related to its biological activity, the formulations were tested in a biological setting for their efficiency in inducing apoptosis. Apoptosis was assessed by the occurrence of cells with nuclear condensation and fragmentation by Hoechst staining [46]. In order to compare the apoptotic activity of USM[CPT] and USM-PEG[CPT] formulations, stock solutions were prepared, and suitable amounts were taken so that the provided final concentration of CPT was identical to that used in the pure CPT control. According to our results, neither USM nor USM-PEG nanoparticles induce apoptosis in lung cancer cell line cultures H460, while USM[CPT] and USM-PEG[CPT], as well as CPT itself, induced apoptosis (Figure 7). The apoptotic levels obtained with both USM[CPT] and USM-PEG[CPT] formulations are comparable to that obtained with CPT. The slight activity decrease observed in formulations when compared to CPT (about 10–15%) is probably due to the release profile of the drug from the formulations, since an increase of the time during which the cell cultures are incubated with the formulations leads to an increased apoptosis (data not shown).

In summary, two new CPT formulations based on iron oxide superparamagnetic nanoparticles have been described and characterized by FTIR. Both formulations retain the biological activity of CPT and exhibit remarkable cytotoxic activity towards H460 lung cancer cell line cultures. Remarkably, it was found that iron oxide superparamagnetic nanoparticles synthesized by co-precipitation method can be loaded with CPT. By the proposed nanoparticle surface modification procedure with PEG the amount of CPT that can be loaded was greatly enhanced (in effect doubled) with respect to bare USM nanoparticles. No significant difference in the cytotoxic activity was observed among the CPT loaded on either the PEGylated or bare USM magnetic nanoparticles. Nevertheless, a different in vivo behaviour of PEGylated and non-PEGylated USM nanoparticles cannot be ruled out, as longer circulation times are expected for the PEGylated ones. Additionally, we envisage that the developed USM-PEG may form targeted-delivery systems by exploiting the free amine groups on their surface for covalent attachment of targeting cargoes such as antibodies.

## Supporting Information

File 1 General procedures.
