# Supplementary material for: PEGylated versus non-PEGylated magnetic nanoparticles as camptothecin delivery system
Source: Beilstein J Nanotechnol. 2014 Aug 19;5:1312–9. doi: 10.3762/bjnano.5.144 (PMC4168894; doi:10.3762/bjnano.5.144)
Supplement: File 1 — General procedures. [file Beilstein_J_Nanotechnol-05-1312-s001.pdf]

Supporting Information

for

PEGylated versus non-PEGylated magnetic nanoparticles as Camptothecin delivery system

Paula M. Castillo[1,2], Mario de la Mata[3], Maria F. Casula[1], José A. Sánchez-Alcázar[3] and Ana P. Zaderenko[2]*

Address: [1]INSTM and Dipartimento di Scienze Chimiche e Geologiche. Università di Cagliari, Italy; [2]Departamento de Sistemas Físicos, Químicos y Naturales. Universidad Pablo de Olavide, Sevilla, Spain and [3]Centro Andaluz de Biología del Desarrollo (CABD-CSIC-Universidad Pablo de Olavide), Sevilla, Spain

Email: Ana Paula Zaderenko* - apzadpar@upo.es

*Corresponding author

General procedures

Experimental: Materials and Methods

Materials

All chemicals are of reagent grade and have been used without further purification: iron (II) chloride tetrahydrate ($FeCl_2 \cdot 4H_2O$, ≥99%), iron (III) chloride hexahydrate ($FeCl_3 \cdot 6H_2O$, 97%), ammonia (28-30%), succinic acid, and poly (ethylene glycol)-bis-(3-aminopropyl)-terminated (PEG; 1500 g/mol) were purchased from Sigma-Aldrich. N-(3-dimethylaminopropyl)-N´-ethylcarbodiimide hydrochloride (EDC) and N-hydroxysuccinimide (NHS) were purchased from Alfa Aesar. Camptothecin (CPT) was purchased from Alexis Biochemicals. As a reference material, magnetite (Fe_3O_4, 99.997%) was purchased from Alfa Aesar. Non-small lung cancer cell line, H-460 was a gift from Dr. P.J. Woll (CRC Department of Clinical Oncology, City Hospital, Nottingham, UK). Cells H460 were cultured in RPMI 1640 by Biowhittaker. Water was purified using a Milli-Q reagent-grade water system from Millipore. Buffers were prepared according to standard laboratory procedures.

Synthesis

USM nanoparticles. Magnetic iron oxide nanoparticles (USM: UltraSmall Magnetite [Fe_3O_4] nanoparticles) were synthesized by iron co-precipitation under alkaline conditions as described by Bee et al. (1995) with slight modifications (Casula et al. 2011). Briefly, two aqueous solutions containing stoichiometric amounts of ferrous chloride (0.5 mL, 2M) and ferric chloride (2 mL, 1M) were mixed together under magnetic stirring. The co-precipitation reaction was then initiated by dropping into the mixture an aqueous solution of ammonia (25 mL, 1M, addition time 30 minutes), which turns the solution black, indicating the formation of USM nanoparticles. After addition, stirring is maintained for one hour at room temperature, and USM nanoparticles are recovered and purified from reaction by-products and excess

ammonia by means of three steps of magnetic separation, removal of the supernatant and washing with water to obtain USM as a black powder.

USM-Suc nanoparticles. Succinic acid (83 mg) was added to an aqueous solution of USM nanoparticles (12 mL, 10.6 mg) and the reaction was allowed to proceed for 24 hours under vigorous stirring. USM-Suc nanoparticles were recovered and purified by means of three steps of magnetic separation, removal of the supernatant and washing with water to obtain 15.5 mg of USM-Suc as a black powder.

USM-PEG nanoparticles. EDC (12 mg, 0.06 mmol) and NHS (18 mg, 0.15 mmol) were added to an aqueous solution of USM-Suc (2.5 mL, 6 mg) and the mixture was stirred for 30 minutes at room temperature. Afterwards, amino-terminated PEG (333 mg) was added to the reaction mixture and stirred for 24 hours at room temperature. Finally, USM-PEG nanoparticles were recovered and purified by means of three steps of magnetic separation, removal of the supernatant and washing with water to obtain 7.5 mg of USM-PEG as a black powder.

CPT loading

Loading of CPT in USM and USM-PEG nanoparticles was done by direct incubation of the drug in aqueous dispersions of the nanoparticles. To determine the *maximum loading capacity* of USM and USM-PEG nanoparticles, increasing volumes (from 1 to 25 µL in 1 µL steps) of a solution of CPT in dimethylsulfoxide (DMSO, 28.7 mM) were slowly added to an aqueous solution of USM or USM-PEG nanoparticles (700 µL, 1 mg/mL). After addition, the sample was gently vortexed and the mixture was incubated for one hour under vigorous oscillating stirring. Afterwards, an aqueous solution of Bovine Serum Albumin (BSA, 63 µL/mL, 10%) was added to the mixture,

which was incubated for an additional hour. CPT-loaded nanoparticles, USM[CPT] and USM-PEG[CPT], were recovered and purified by means of three steps of magnetic separation, removal of the supernatant and washing with PBS (pH 7.4).

CPT content in nanoparticles was estimated by an indirect method. The supernatants and washing solutions obtained during the CPT loading steps were collected and assayed by UV-Vis spectroscopy to determine the amount of CPT that was not loaded. A standard curve of CPT was prepared under identical conditions, and free CPT in supernatants was determined by measuring CPT signal at 370 nm. Loading capacity (LC) was calculated according to Eq. (1).

Characterization

Transmission Electron Microscopy (TEM) micrographs were recorded on a JEOL (Tokyo, Japan) 200CX microscope operating at 200 kV. Prior to observations, the water-based magnetic suspension was dropped on a carbon-coated copper grid and dried at room temperature.

X-Ray Diffraction Analysis (XRD) was recorded on a Seifert X3000 diffractometer, using Cu K_α radiation, equipped with a graphite monochromator on the diffracted beam. The samples were deposited and dried on silicon zero-background sample holders. Phase identification was performed according to the Powder Diffraction File database, ICDD International Centre for Diffraction Data, 1601 Park Lane, Swarthmore, USA. The average crystal size was determined by line profile

broadening corrected for instrumental broadening according to the Scherrer equation.[1]

UV-visible spectra were recorded on an Ocean Optics spectrometer equipped with a HR4000 detector.

Fourier Transform Infrared (FTIR) spectra were recorded on a Bruker IFS 66/s spectrometer equipped with a DTGS detector (Billerica, MA, USA). Samples were prepared by dropping and drying, at room temperature, 100 μl of the water-based magnetic suspension on a silicon wafer. The spectra were obtained from averaging 100 scans with a scan frequency of 2.5 Hz and 3 cm^{-1} resolution.

The magnetic parameters of the USM dry nanoparticles were investigated by Superconducting Quantum Interference Device (SQUID) magnetometry, on a Quantum Design MPMS SQUID magnetometer equipped with a superconducting magnet capable of producing fields up to 50 kOe. The Zero-Field-Cooled (ZFC) magnetization was collected by first cooling the sample down to 5K under zero magnetic field and then increasing the temperature up to 325 K in an applied field of 25 Oe, whereas Field-Cooled (FC) curves were recorded by cooling the sample in the same field of 25 Oe.

The isothermal hysteretic behaviour of the USM nanoparticles was investigated by recording the field dependence of the magnetization (hysteresis loop) up to ±50 kOe at 5 K.

[1] Klug HP and Alexander LE (1974). X-ray Diffraction Procedures. Wiley, New York.

Cell culture

Non-small lung cancer cell line H460 was cultured in RPMI medium supplemented with penicillin, streptomycin and 10% Fetal Bovine Serum (FBS) at 37 ºC until 80% confluence.

Stock dispersions of USM[CPT] and USM-PEG[CPT] were prepared in PBS (pH 7.5) with a CPT concentration of 5 mM, and 1 µL of this dispersion was added to H460 cells previously cultured (confluent; 1mL final volume, 5 µM final CPT concentration). Cells with nanoparticles and references were incubated at 37 °C for 48 h. For comparison, the following H460 cell culture samples were incubated in the same way: a control that contained neither nanoparticles nor CPT, nanoparticles without CPT (USM and USM-PEG) and 1 µL CPT (5 mM in DMSO, 5 µM final CPT concentration in cell culture).

Fluorescence studies and Analysis of apoptosis. H460 cells were grown on 1 mm (Goldseal No.1) glass coverslips for 24–48 h in RPMI containing 10% FBS. Depending on the sample, cells are either treated with CPT, CPT-loaded nanoparticles, or nothing at all and left for 48 h. At that stage, cells were rinsed once with PBS, fixed in 3.8% paraformaldehyde for 5 min at room temperature, and permeabilized in 0.1% saponin for 5 min. Cells were subsequently stained with Hoechst 33342 (1 mg/mL and washed with PBS (three 5 min washes). Apoptotic cell nuclei were studied by fluorescence microscopy using an Upright Fluorescence Microscopy Zeiss Axio Imager M2 (Carl Zeiss MicroImaging GmbH, Jena Germany). Lens: 20x air. Camera: AxioCam. Coverslips were mounted onto microscope slides using Vectashield Mounting Medium (Vector Laboratories, Burlingame, CA, USA). MRmFilter Configuration: Apotome, Software: AxioVision 4.7 was used to visualize

the apoptotic effect in cell lines after being incubated with nanoparticles and cell viability quantified. Image J software was used for image analysis and cell count.

Statistical analyses. All results are expressed as mean ± standard deviation. Two-tail Student's *t*-tests (paired and unpaired, according to samples) or ANOVA variance analyses were used as appropriate for statistical analysis. Only P-values <0.05 were considered as statistically significant.
